# Purkinje Cell Degeneration and Motor Coordination Deficits in a New Mouse Model of Autosomal Recessive Spastic Ataxia of Charlevoix-Saguenay

**DOI:** 10.3389/fnmol.2017.00121

**Published:** 2017-05-01

**Authors:** Man Ding, Chao Weng, Shanghua Fan, Qian Cao, Zuneng Lu

**Affiliations:** Department of Neurology, Renmin Hospital of Wuhan UniversityWuhan, China

**Keywords:** Ankfy1, ARSACS, neurodegenerative disease, NTFs, behavior, apoptosis

## Abstract

Autosomal recessive spastic ataxia of Charlevoix-Saguenay (ARSACS) is an early-onset neurodegenerative disorder. In 2007, a novel locus, SAX2, which is located on chromosome 17p13 and contains 3 genes, ankyrin repeat and FYVE domain-containing 1 (*ANKFY1*), β-arrestin 2 (*ARRB2*) and kinesin family member 1C (*KIF1C*), was linked to ARSACS. We generated Ankfy1 heterozygous (Ankfy1/+) mice to establish an animal model and examine the pathophysiological basis of ARSACS. The transgenic mice displayed an abnormal gait with progressive motor and cerebellar nerve dysfunction that was highly reminiscent of ARSACS. These clinical features were accompanied by an early-onset and progressive loss of Purkinje cells, followed by gliosis. Additionally, the loss of Ankfy1 function resulted in an abnormal expression of neurotrophic factors (NTFs) in the Ankfy1/+ mouse cerebellum. Moreover, Purkinje cells cultured from neonatal Ankfy1/+ mice exhibited a shorter dendritic length and decreased numbers of dendritic spines. Importantly, cerebellar Purkinje cells from Ankfy1/+ mice and cells transfected with a lentiviral Ankfy1 shRNA underwent apoptosis. We propose that transgenic Ankfy1/+ mice are a useful model for studying the pathogenesis of ARSACS and for exploring the molecular mechanisms involved in this neurodegenerative disease.

## Introduction

Hereditary ataxias are classified as autosomal dominant, autosomal recessive, X-linked and mitochondrial based on their mode of inheritance. Autosomal recessive cerebellar ataxias (ARCAs) are heterogeneous and complex neurodegenerative disorders. Early-onset ARCAs represent a subgroup for which several disease genes have been identified as playing a role in processes such as DNA damage responses, DNA repair, DNA transcription, DNA replication, RNA processing, protein folding and modification and neurotransmitter metabolism (Fogel and Perlman, 2007). ARCAs are also a heterogeneous group of disorders that primarily include Friedreich’s ataxia, ataxia telangiectasia, ataxia with vitamin E deficiency, autosomal recessive spastic ataxia of Charlevoix-Saguenay (ARSACS), abetalipoproteinemia and ataxia with oculomotor apraxia types 1 and 2 (Di Donato et al., 2001). Due to the clinical and genetic heterogeneity of these disorders, clinicians may initially be unable to differentiate ARCAs from other forms of ataxia. The key prototypic feature shared by all these categories is spinocerebellar ataxia involving the cerebellum, brainstem, or spinocerebellar long tracts. Autosomal dominant cerebellar ataxias (ADCAs) may also have diverse associated neurological features, including retinopathy, optic atrophy, extrapyramidal or pyramidal signs, peripheral neuropathy, cognitive impairment, or epilepsy (Schöls et al., 2004). In contrast, ARCAs are primarily characterized by progressive cerebellar dysfunction, pyramidal signs such as spasticity with hyperreflexia, and peripheral sensorimotor neuropathy with amyotrophy (El Euch-Fayache et al., 2003; Schöls et al., 2004). ARCAs were first identified in northeast Canada (Engert et al., 2000) and have more recently been described in China, Northeast Lebanon, Turkey and Morocco (Jobling et al., 2015; van Schil et al., 2015; Duan et al., 2016; Nguyen et al., 2016).

A 2007 study of 15 families with 34 affected members with ataxia and pyramidal signs or spasticity linked ARSACS to a 6.1-cM region on chromosome 17p13 named SAX2 that contains the genes ankyrin repeat and FYVE domain-containing 1 (*ANKFY1*), β-arrestin 2 (*ARRB2*) and kinesin family member 1C (*KIF1C*, Bouslam et al., 2007). No mutations were detected in the coding exons of these genes, but rearrangements or mutations in their regulatory regions or in non-described exons cannot be excluded (Bouslam et al., 2007). The *ARRB2* gene encodes the β-arrestin 2 protein, which regulates agonist-mediated G-protein coupled receptor (GPCR) signaling by mediating both receptor desensitization and resensitization processes (Shukla et al., 2011). β-arrestin 2-deficient mice have been shown to exhibit impaired memory reconsolidation in the object recognition, Morris water maze and cocaine-conditioned place preference paradigms (Liu et al., 2015). The KIF1C protein is expressed in all tissues examined, with the highest expression in the heart and skeletal muscle (Dorner et al., 1998).

The *ANKFY1* gene was first described by Ito et al. (1999). The human *ANKFY1* gene encodes Ankfy1, a multimodular protein consisting of 1166 amino acids that exhibits 84.9% identity to the mouse homolog, which is highly expressed in the adult brain and spinal cord (Kuriyama et al., 2000). From the N- to C-terminus, Ankfy1 contains a BTB/POZ domain that acts as a specific protein-protein interaction interface and mediates protein oligomerization, 21 ankyrin repeats that specifically bind to proteins or macromolecules, and a FYVE-finger domain that encodes a double zinc finger protein in combination with phosphatidylinositol 3-phosphate (PI(3)P), which may be involved in vesicle or protein transport (Kuriyama et al., 2000). The nature of these key features suggests that Ankfy1 may perform special functions. Ankfy1 is a PI(3)P-binding Rab5 effector that is predominantly involved in early endosome fusion and macropinocytosis (Schnatwinkel et al., 2004). In addition, Ankfy1 has important roles in the internalizing and trafficking activated tyrosine kinase receptors, such as platelet-derived growth factor receptor β (PDGFRβ; Nehru et al., 2013).

Previously, we established an Ankfy1 transgenic mouse model in which the gene was deleted from exon 5. Unfortunately, we did not obtain knock-out mice due to embryonic lethality. However, in the current study, Ankfy1/+ heterozygous mice exhibited motor deficits. Our studies confirm that motor behavior abnormalities are expressed as impaired motor activity, coordination and skill. Ankfy1 depletion damages the cerebellum, and the neurodegeneration leads to the progressive loss of Purkinje cells. In addition, primary cultured Purkinje cells from Ankfy1/+ mice exhibited shorter dendrites with reduced numbers of dendritic branches. The expression of representative genes, including those encoding neurotrophic factors (NTFs) such as brain-derived neurotrophic factor (BDNF), neurotrophin-3 (NT-3) and mesencephalic astrocyte-derived neurotrophic factor (MANF), was detected to study the roles of Ankfy1 in organ development and maintaining function and were found to be altered. Due to the characteristics of this mutation, the ideal model would be one in which Ankfy1 levels are reduced but not completely eliminated.

## Materials and Methods

### Generation of Mutant Ankfy1 Mice

All mice were bred and maintained in the Center for Animal Experiments/Animal Biosafety Level-III Laboratory in Wuhan University under specific pathogen-free conditions in accordance with the National Institutes of Health Guide for the Care and Use of Laboratory Animals (National Research Council Publication, 1996 edition) and the protocol was approved by the Institutional Animal Care and Use Committee of Wuhan University. The transgenic mice were generated on the C57BL/6 background. Ankfy1 mutants were purchased from BayGenomics (UC Davis, Davis, CA, USA) and contained the gene-trap vectors that were randomly inserted into intron 4. Genomic DNA samples from tail biopsies were genotyped using PCR to identify founders. The control genotyping reaction yielded a 384-bp band using the following primers: forward, 5′-AATGGCCCCCAACTTTATTT-3′ and reverse, 5′-CAAAGTGTGTGCCACCAATC-3′. The band for the mutant Ankfy1 allele was obtained by PCR of genomic DNA samples using primers for β-geo (forward, 5′-CAAATGGCGATTACCGTTGA-3′ and reverse, 5′-TGCCCAGTCATAGCCGAATA-3′).

### Mouse Behavioral Tests

Behavioral tests were performed during the diurnal period in groups of 6 Ankfy1/+ transgenic mice and wild-type (WT) littermates (*n* = 8–13 per genotype) per cage. All behavioral tests were started at 4 weeks of age and were conducted until the mice reached 50 weeks of age.

#### Rotarod Test

Mice were tested using a rotarod apparatus (TSE systems, Bad Homburg, Germany) to evaluate their motor performance. They were evaluated monthly on the rotarod. The protocol consisted of 3 days of training at a constant speed (10 rpm) for 10 min in three trials, with a 10-min interval between each trial. On the fourth day, the animals were subjected to four trials on an accelerating rod (4–30 rpm, 5 min) with a 10-min interval between each trial. Rotarod performance was quantified by recording the latency to fall.

#### Footprint Pattern

The footprint test was used to evaluate the animals’ gaits. The hind and fore paws of the mice were coated with non-toxic blue and red paints, respectively, to obtain footprints. A clean sheet was placed on the floor of the runway for each run. The animals were then allowed to walk along a 100-cm long × 10-cm wide × 10-cm high corridor in the direction of an enclosed black box. Each animal was allowed to achieve one valid trial at each age. The footprint patterns were analyzed for six step parameters (all measured in cm): the front- and hind-base width and length. For each step parameter, three values were measured for three consecutive steps, with the exclusion of the first four steps to allow for habituation. The footprinting patterns of Ankfy1/+ (*n* = 8) and WT controls (*n* = 10) were classified at each time point for six consecutive steps (0 = absent/mild, up to three steps; 1 = moderate, more than three steps out of six; 2 = severe, all steps out of six) to evaluate the severity of foot dragging at each age. A toe drag was counted when the ink streak from a hind paw was longer than one paw length and was located between two hindlimb footprints on the same side.

### *In Situ* Hybridization

Isolated mouse brains and spinal cords were rapidly frozen in 100% optimum cutting temperature (OCT) compound (Tissue-Tek Sakura Finetek USA, Inc., Torrance, CA, USA) in a dry ice-hexane bath. Frozen brains and spinal cords were sectioned at 14-μm thickness (Thermo Fisher Scientific; Walldorf, Germany) and mounted on aminopropylsilane (APS)-coated slides. Sections were fixed with 4% paraformaldehyde for 10 min. Basic proteins were acetylated with 0.25% acetic anhydride in 0.1 M triethanolamine (pH 8.0). Both before and after this process, the slides were rinsed with 1× diethylpyrocarbonate (DEPC)-treated PBS. Pre-hybridization was performed in hybridization buffer (50% deionized formamide, 10% dextran sulfate, 5× Denhardt’s solution, 5× saline-sodium citrate (SSC; 1× SSC, 0.15 M NaCl and 15 mM sodium citrate), 0.25 mg/ml tRNA from baker’s yeast, 0.5 mg/ml boiled DNA from herring sperm) without riboprobes for 20 min at room temperature. Hybridization was performed overnight at 65°C in hybridization buffer with 0.25 mg/ml digoxigenin (DIG)-labeled riboprobes, which were denatured at 95°C for 5 min. At the end of the incubation, the sections were sequentially rinsed with 2× SSC and 0.2× SSC at 67°C. The sections were then blocked with 10% sheep serum for 1 h at room temperature. The sections were incubated with an alkaline phosphatase-conjugated sheep anti-DIG antibody (Roche, Germany; 1:2000) in 10% sheep serum overnight at 4°C. Staining was visualized using a nitroblue tetrazolium (6.6 μl/ml) and 5-bromo-4-chloro-3-indolyl-phosphate (3.3 μl/ml) staining solution.

### Cell Culture, Plasmids and Transfection

The cerebella of neonatal (postnatal day 0 or 1) WT (C57BL/6) or Ankfy1/+ mice were dissected in ice-cold PBS. After removing the meninges and mincing the tissues with eye scissors, the specimens were incubated with 4 U/ml papain (Sigma, St. Louis, MO, USA), 20 U/ml DNase I (Sigma, St. Louis, MO, USA) and 5 mM cysteine in PBS for 30 min at 37°C. Foetal bovine serum was added to stop the proteolytic reaction, and the mixture was strained through a 40-μm nylon mesh filter (Falcon 2340, Becton Dickinson, Franklin Lakes, NJ, USA). After centrifugation at 1000 rpm for 1 min, the tissues were triturated with a pipette and resuspended in stop medium at a final concentration of 5 × 10^6^ cells/ml. Then, the tissues were plated on coverslips (14 mm in diameter) coated with poly-D-lysine (Sigma, St. Louis, MO, USA). After 1–2 h of incubation, 1 ml of Dulbecco’s Modified Eagle’s Medium/F12 (HyClone) containing 1.4 mM L-glutamine, B27 supplement (50×; Gibco) and penicillin-streptomycin was added to each culture dish. The cultures were incubated at 37°C in 5% CO_2_/95% air. The medium was changed once a week.

A172 cells were cultured in Dulbecco’s Modified Eagle’s Medium (HyClone) supplemented with 10% foetal bovine serum. The human *Ankfy1* cDNA was obtained by RT-PCR and inserted into the pLKO.1 vector. Additionally, green fluorescent protein (GFP) was cloned into the pLKO.1 lentiviral backbone in place of the puromycin resistance gene. The following primers were used to generate the Ankfy1 shRNA: sense 5′-CCGGGCAGTGCAAACAACTAGATTTCTCGAGAAATCTAGTTGTTTGCACTGCTTTTTG-3′, antisense 5′-AATTCAAAAAGCAGTGCAAACAACTAGATTTCTCGAGAAATCTAGTTGTTTTGCACTGC-3′. The following primers were used to generate the negative control (NC) shRNA: sense 5′-CCGGCCTAAGGTTAAGTCGCCCTCGCTCGAGCGAGGGCGACTTAACCTTAGGTTTTTG-3′, antisense 5′-AATTCAAAAACCTAAGGTTAAGTCGCCCTCGCTCGAGCGAAGGGCGACTTAACCTTAGG-3′. The shRNAs were inserted into the pLKO.1 vector at the *AgeI* and *EcoRI* restriction sites. HEK293T cells were transfected with the Ankfy1 or NC shRNA vector, as well as psPax2 and pMD2G vectors in a 10-cm dish using the Neofect™ DNA transfection reagent (Neofect (Beijing) Biotech Co., Ltd., China). The culture medium was collected 48 h after transfection. For experiments using the infected A172 cell line, the virus from the conditioned culture medium was used to infect A172 cells according to the instructions provided with the Neofect™ DNA transfection reagent. The NC and Ankfy1 shRNA were generated in parallel. Images of the fluorescent proteins were acquired using a Nikon microscope (Nikon, Japan), and fluorescence was quantified using Adobe Photoshop and ImageJ (NIH) software.

### Immunostaining

Twenty-four-week-old WT and Ankfy1/+ littermate mice (*n* = 4 or 5 for each group) were deeply anesthetized and sequentially perfused with PBS and 4% paraformaldehyde. The brains were removed and post-fixed with 4% paraformaldehyde overnight, followed by dehydration with 20% sucrose. The brains were then embedded in OCT (SAKURA, USA). Slides containing 14-μm frozen sections and primary Purkinje cells from WT or Ankfy1/+ mice were steamed for antigen retrieval and then incubated with mouse anti-calbindin D-28k (cb300, Swant, Switzerland; 1:100) or goat Ankfy1(D-15; sc-160136, Santa Cruz Biotechnology; 1:100) antibodies. Then, Cy3-conjugated rabbit anti-goat (AS015, ABclonal, UK; 1:500), FITC-conjugated goat anti-mouse (SA00003-1, Proteintech, Chicago, IL, USA; 1:500), FITC-conjugated rabbit anti-goat (A22130, Abbkine, Redlands, CA, USA; 1:500) or Alexa Fluor 555-conjugated goat anti-mouse (A21422, Invitrogene, USA; 1:500) secondary antibodies were applied. Samples of the cerebellum, substantia nigra and vestibular nuclei were incubated with the mouse glial fibrillary acidic protein (GFAP)-Cy3 (C9205, Sigma-Aldrich, USA; 1:200) antibody and then mounted with DAPI-Fluoromount G (Southern Biotech, USA). For the analysis of brain morphology, we performed hemeatoxylin and eosin (H&E) and Eriochrome cyanine staining. The stereological analysis of GFAP-positive cells was performed using Adobe Photoshop software. Quantitative analyses of the number of cerebellar Purkinje cells, the thickness of the cerebellar molecular layer or medulla, and the thickness of the corpus callosum were performed using ImageJ software (NIH). Measurements were recorded from randomly selected regions. The total area covered by the dendrite tree of randomly selected Purkinje cells (*n* = 10) from each experiment was determined by tracing the outline of the cell body and the dendrite branches followed by analysis using ImageJ (NIH), MATLAB (MathWorks) and NeuroStudio software (Langhammer et al., 2010) to quantify the number and branching of primary Purkinje cell dendrites.

The TUNEL procedure was performed using a TUNEL Apoptosis Detection Kit (Alexa Fluor 647; 40308, Yeasen, ShangHai, China) according to the manufacturer’s instructions. Five sections were randomly selected from the cerebella of 6-month-old Ankfy1/+ and WT mice. The number of TUNEL-positive nuclei was counted in each image. Values obtained from five sections of each cerebellum were averaged. Visualization and quantification were performed by an observer who was blinded to the groups to avoid bias.

### Western Blotting

For each blot, tissues from mutant or control mice were rapidly dissected and then placed in radioimmunoprecipitation (RIPA) buffer containing protease inhibitors before homogenization. First, the culture medium was aspirated and the cells were then washed with ice-cold PBS. Subsequently, the cells were homogenized with RIPA buffer. The tissues or cells were then sonicated for 10 s. Equal amounts of protein were separated on SDS polyacrylamide gels and transferred to nitrocellulose membranes. Blots were blocked with 5% non-fat dry milk or 5% BSA in Tris-buffered saline (10 mM Tris, pH 7.6 and 150 mM NaCl) containing 0.1% Tween 20 for 1 h at room temperature. Membranes were incubated with specific antibodies overnight at 4°C. Mouse Ankfy1(B-6; sc-393353, Santa Cruz Biotechnology; 1:100), rabbit β-actin (10494-1-AP, Proteintech, Peking, China; 1:10,000), rabbit caspase3 (9662, Cell Signaling Technology, Danvers, MA, USA; 1:1000), rabbit cleaved caspase3 (AF7022, Affinity Biosciences, USA; 1:1000), rabbit caspase9 (AF6348, Affinity biosciences, USA; 1:1000), rabbit cleaved caspase9 (AF5244, Affinity biosciences, USA; 1:1000), mouse monoclonal caspase8 (66093-1-Ig, Proteintech, Peking, China; 1:800), Akt (9272, Cell Signaling Technology, Danvers, MA, USA; 1:1000) and p-Akt (Ser473; 9271, Cell Signaling Technology, Danvers, MA, USA; 1:1000) antibodies were used. Blots were then incubated with specific secondary antibodies. Immunoreactive proteins were detected with an enhanced chemiluminescence detection system. The relative density of the protein bands was quantified by densitometry using ImageJ software (NIH).

### RT-qPCR

Animals were euthanized by decapitation, and the cerebellum was collected, immediately snap frozen and stored at −80°C. Total RNA samples from mouse cerebella and brains obtained at 14, 21 and 30 days of age (*n* = 10–11) were extracted using TRIzol reagent (Invitrogen) according to the manufacturer’s protocol. Total RNA was first treated with DNase I (Fermentas) and converted to first-strand cDNA using the RevertAid First Strand cDNA Synthesis Kit (K1622, Thermo Scientific) according to the manufacturer’s guidelines. Glyceraldehyde 3-phosphate dehydrogenase (GAPDH) was used as an internal control. In addition, we analyzed the expression levels of the BDNF, MANF, NT-3, retinoic acid receptor-related orphan receptor α (RORα), GFAP and myelin basic protein (MBP) genes using RT-qPCR and the following primers: mouse BDNF forward primer (5′-GAAGGGTTTCTTACCTGGCGAC-3′), reverse primer (5′-AGCCCT AGCACAAAAAGTTCCC-3′); mouse MANF forward primer (5′-CGGTACTTCACCTCATCTCCTG-3′), reverse primer (5′-AACCTACAGACAGGCATCTTGG-3′); mouse NT-3 forward primer (5′-ACTCCAGAAGCTGACCATCAAG-3′), reverse primer (5′-CTCCAGTCTCAATTCCCGAAGG-3′); mouse RORα forward primer (5′-CTTCCCCTACTGTTCCTTCACC-3′), reverse primer (5′-CACATCACCTCTCTCTGCTTGT-3′); mouse MBP forward primer (5′-CAAGGAAGGGAGGAAGAGA CA-3′), reverse primer (5′-CGGGATTAAGAGAGGGTCTGCT-3′); mouse GFAP forward primer (5′-GTGGGCAGGTGGGAGCTTGATTCT-3′), reverse primer (5′-CTGGGCGGCCTGGTATGACA-3′); mouse Ankfy1 forward primer (5′-ATGGTTGCGATGCTACATGCTG-3′), reverse primer (5′-GTCTGTCCATCCCTTGCTTCCT-3′); and mouse GAPDH forward primer (5′-AGGTCGGTGTGAACGGATTTG-3′), reverse primer (5′-GGGGTCGTTGATGGCAACA-3′). We analyzed the expression levels of the Ankfy1 mRNA in A172 cells with RT-qPCR using the following primers: human Ankfy1 forward primer (5′-AGCCTCAAAGATTCCCGAGACC-3′), reverse primer (5′-CGTCCTGAGTCCTGCTGACATT-3′) and human GAPDH forward primer (5′-GGGAGCCAAAAGGGTCATCA-3′), reverse primer (5′-TGATGGCATGGACTGTGGTC-3′). Data were analyzed using the comparative threshold cycle (C_t_) method, which for accuracy requires similar amplification efficiencies between the target and the internal control gene. Amplification efficiencies of the target genes and the internal control gene were validated by determining the C_t_ slope for serially diluted template cDNAs. Data are expressed as fold changes.

### Statistical Analysis

For mouse behavioral data, each group consisted of at least six animals. For immunostaining, western blot analysis, RT-qPCR, or other biochemical assays, the data were generated from three or more experiments. The data were subjected to the non-parametric Mann-Whitney U-test when variables were non-continuous or when a continuous variable did not display a normal distribution (Kolmogorov-Smirnov test *p* < 0.05). Continuous variables with normal distributions (K-S test *p* > 0.05) were calculated using Student’s *t*-test or ANOVA. *P* < 0.05 was considered statistically significant throughout the study.

## Results

### Generation of Ankfy1 Transgenic Mice

**Figure 1:**
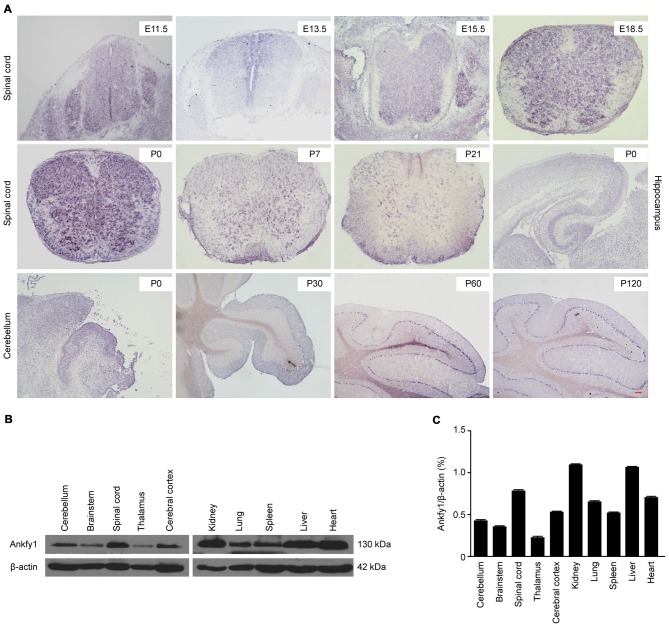
**Expression profiles of Ankfy1. (A)** *In situ* hybridization studies confirm that Ankfy1 is expressed in the spinal cord (E11.5, E13.5, E15.5, E18.5, P0, P7 and P21), hippocampus (P0) and cerebellum (P0, P30, P60 and P120) of wild-type (WT) mice. The scale bar represents 200 μm. **(B)** Expression levels of the Ankfy1 protein in 10 adult mouse tissues were determined using western blotting. **(C)** Representative results are shown, and graphs present the ratio of Ankfy1 to β-actin. The data are presented as the means from three independent experiments.

**Figure 2:**
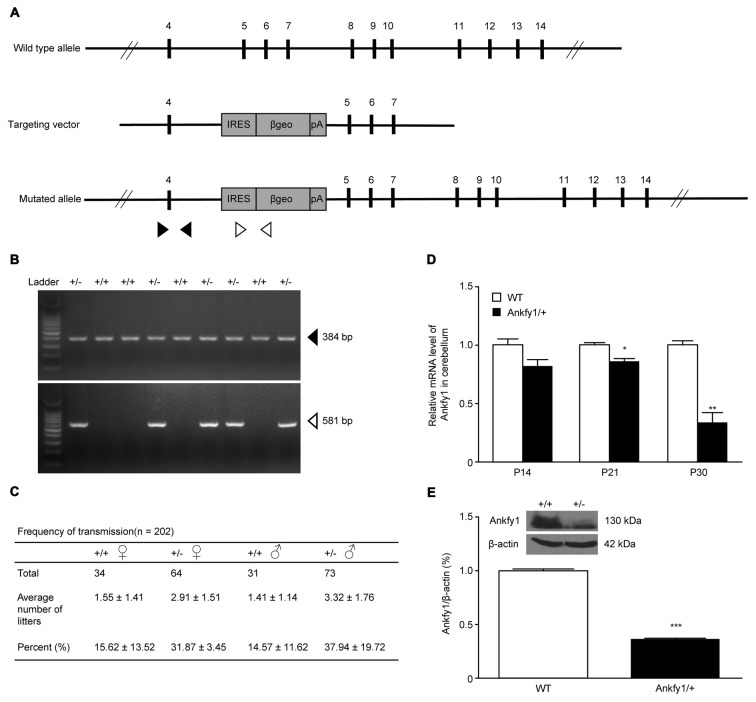
**Generation of Ankfy1/+ mutant mice. (A)** Schematic representation of the targeting constructs. The internal ribosome entry site (IRES)-βgeo-polyA cassette was inserted into intron 4 of the *ANKFY1* locus. The IRES-βgeo-polyA cassette is presented as a gray box. It contains the fusion gene *βgeo*(*lacZ-neo*) placed under the control of an IRES. Arrowheads indicate the primers used for protein coupled receptor (PCR). **(B)** PCR analyses. Solid arrows indicate bands corresponding to the WT locus, whereas open arrows indicate bands corresponding to the targeted locus. +/+, WT mice; +/−, heterozygous mutant mice. **(C)** Birth rates of WT mice were approximately half of the expected heterozygous Mendelian ratios (1:2). **(D)** RT-qPCR analyses of Ankfy1 mRNA levels in the cerebella of WT and Ankfy1/+ mutant mice (*p < 0.05, **p < 0.01). **(E)** Cerebella from different adult mice were immunoblotted with a polyclonal mouse antibody against Ankfy1. The levels of the β-actin protein in the lysates are shown as an internal control (***p < 0.001). The data represent means ± standard errors of the means (SEM).

ARSACS is considered a neurodegenerative disease caused by mutations in the SACS gene, located on chromosome 13q12.12. In 2007, three genes located on human chromosome 17p13, *ANKFY1*, *ARRB2* and *KIF1C*, were reported to be associated with ARSACS (Bouslam et al., 2007). Ankfy1 is ubiquitously expressed in human tissues (Ito et al., 1999). *In situ* hybridization revealed that the Ankfy1 mRNA was expressed in the spinal cord from the embryonic to postnatal stages, the hippocampus and particularly the cerebellum (Figure 1A). Western blotting revealed the expression of the Ankfy1 protein in different systems (Figures 1B,C). Based on its abundant expression in the central nervous system (CNS), an *ANKFY1* transgenic mouse model was generated in which an IRES-βgeo-polyA selection cassette was inserted into intron 4 using gene targeting (Figure 2A). PCR genotyping with two pairs of primers to detect normal and mutated alleles revealed the presence of the transgene in the offspring of the founders (Figure 2B). Our analysis of the transmission of the transgene revealed an adequate fit to expected Mendelian ratios, but homozygotes were not obtained (Figure 2C), indicating that Ankfy1^−/−^ transgenic mice died during embryogenesis. At the time of birth, no obvious changes in Ankfy1 mRNA expression were detected between WT and Ankfy1/+ transgenic mice. The expression of the Ankfy1 mRNA was decreased in the cerebellum of transgenic mice at P21. Additionally, expression only reached approximately 30% of WT levels at P30 (Figure 2D). Western blots and quantification of the ratio of Ankfy1 to β-actin in the cerebellum of adult WT and Ankfy1/+ transgenic mice also verified the significant loss of Ankfy1 in heterozygotes (Figure 2E). Moreover, the brain, especially the cerebellum, of the Ankfy1/+ mice was smaller than that of the WT mice (Supplementary Figure S1A).

### Neurological Alterations in Ankfy1/+ Mice

**Figure 3:**
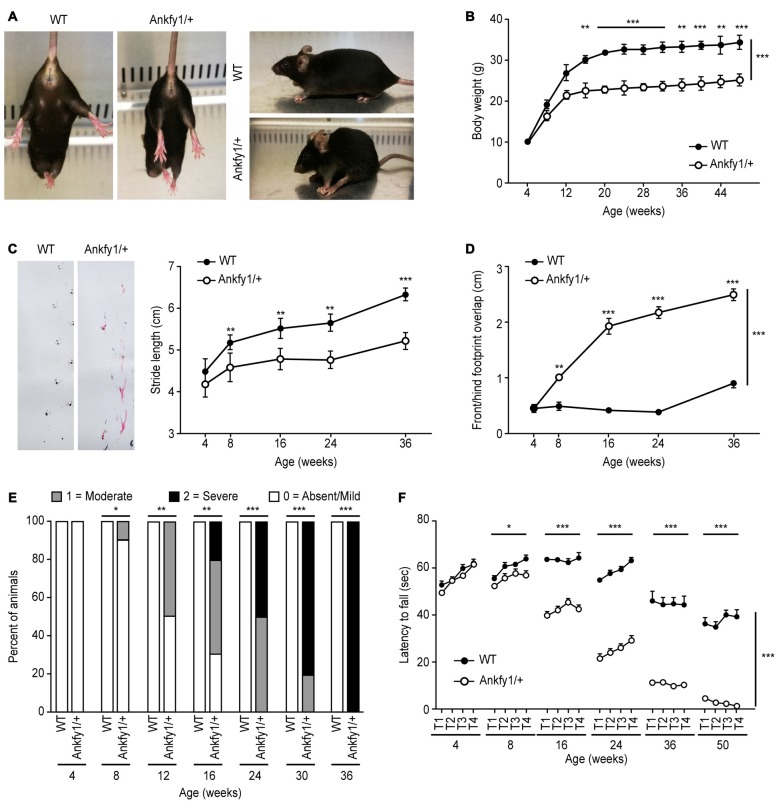
**Impaired motor coordination in Ankfy1/+ mice. (A)** Photographs of Ankfy1/+ mice showing the clasping, spastic paralysis and kyphosis phenotypes, which were not observed in the WT mice. **(B)** Significant differences in body weights were observed between the WT (*n* = 10) and Ankfy1/+ mice (*n* = 9; ***p < 0.001). **(C)** Stride length, **(D)** front/hind footprint overlap and **(E)** foot dragging were evaluated from 4 weeks to 36 weeks of age, and phenotype severity was detected from 8 weeks to 36 weeks of age (*n* = 8–10, **p < 0.05, ***p < 0.001). **(F)** Differences in accelerating rotarod performance observed between WT (*n* = 11) and Ankfy1/+ mice (*n* = 13) were statistically significant (*p < 0.05, ***p < 0.001). The data represent means ± SEM.

Ankfy1/+ transgenic mice suffered from progressive motor deficits and spastic paralysis reminiscent of human ARSACS. Body weights and neurological behavioral tests, including the rotarod test and footprint analyses, were evaluated to gauge disease progression, as described in the “Materials and Methods” Section. We used the mice with neurological deficits to analyze motor coordination and other neurological behaviors. The hind paw clasping phenotype was tested by suspending mice by their tails. Mice that clasped both hind paws tightly to their body were considered to have a complete clasp phenotype. In this study, paw clasping and kyphosis were first observed at approximately 12 weeks of age (Supplementary Figure S1B) in Ankfy1/+ transgenic mice that were suspended by the tail. In addition, spastic hind paw paralysis and kyphosis also occurred in 24-week-old Ankfy1/+ transgenic mice compared with the WT littermates (Figure 3A). We first observed differences in gait between Ankfy1/+ transgenic mice and WT littermates at postnatal day 20, but no differences were observed in rotarod test performance and body weight (data not shown). Ankfy1/+ transgenic mice did not exhibit a significant increase in body weight beginning at approximately 8 weeks, whereas their WT littermates continued gaining weight (Figure 3B). The body weights of these mice were used to reflect disease severity because body weight is more reliably quantifiable than other behavioral phenotypes. According to the footprint analysis, Ankfy1/+ mice had a motor weakness, dragged their hind legs and had a stride length that was much smaller than WT littermates (Figure 3C). Furthermore, Ankfy1/+ mice exhibited an obvious increase in front/hind footprint overlap compared with controls (Figure 3D). Foot dragging was already present in 8-week-old transgenic animals, progressed with age and became increasingly more severe (Figure 3E). The rotarod test is an objective method for quantitatively assessing motor strength and coordination. The ability of mice to remain on a rotarod turning at a uniform speed measures behaviors at the early stage of ARSACS. Consistent with their body weights, 8-week-old Ankfy1/+ mice performed worse in terms of the time spent on a rotating rod, which was significantly shorter than that of age-matched controls (Figure 3F). The Ankfy1/+ mice also displayed tremors, but we did not monitor this abnormality.

### Purkinje Cell Degeneration and Gliosis in Ankfy1/+ Mice

**Figure 4:**
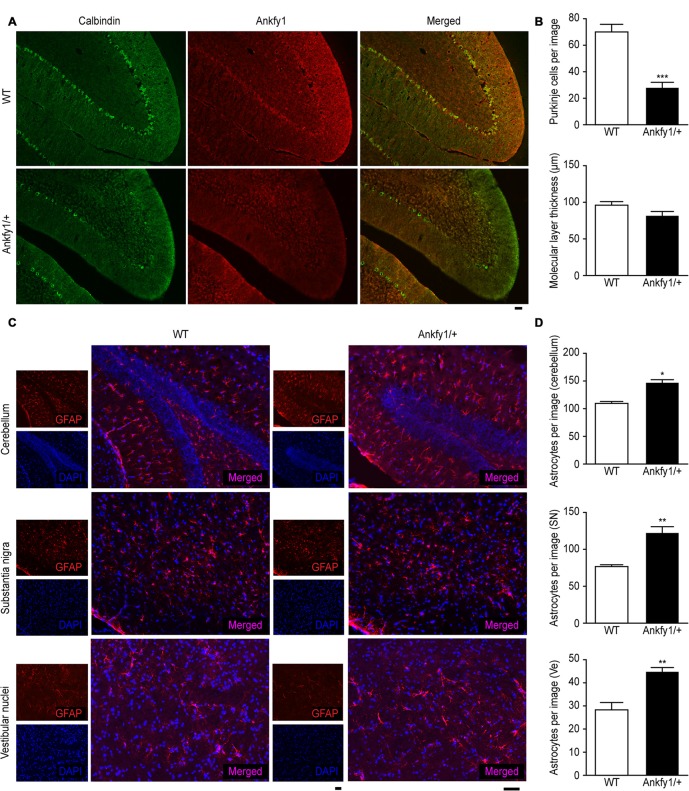
**Abnormal immunostaining patterns in the cerebellum, substantia nigra and vestibular nuclei of Ankfy1/+ mice. (A)** Images of immunofluorescence staining (10× magnification) of WT and Ankfy1/+ mouse cerebella at 6 months of age. Calbindin staining was used to reveal Purkinje cells, and an Ankfy1 antibody was used to detect Ankfy1 expression. The scale bar represents 50 μm. **(B)** Quantitative assessment of the number of Purkinje cells labeled with the anti-calbindin antibody per image field and the thickness of the molecular layer (10×, *n* = 10, ***p < 0.001). **(C)** Images of immunofluorescence staining of the cerebellum, substantia nigra and vestibular nuclei of WT and Ankfy1+ mice at 6 months of age. Glial fibrillary acidic protein (GFAP) staining was used to reveal astrocytes, and DAPI staining was used to detect cell nuclei. The scale bar represents 50 μm. **(D)** Quantitative analysis of the number of astrocytes in WT and Ankfy1/+ mice (20×, *n* = 7–9, *p < 0.05, **p < 0.01). The data represent means ± SEM.

Immunostaining with an antibody to calbindin, a specific Purkinje cell marker, revealed that Ankfy1/+ mice exhibited severe Purkinje cell loss compared with 6-month-old WT mice, although the thickness of the molecular layer was not changed (Figures 4A,B). These results were confirmed by performing conventional H&E staining of the cerebellar cortical region of the mutants from P14 to P60. A marked reduction in the number of Purkinje cells was observed beginning at P60, and the molecular layer was not decreased (Supplementary Figures S1C,D).

In addition, 24-week-old Ankfy1/+ transgenic mice displayed increased numbers of astrocytes, as indicated by GFAP immunostaining in specific areas, such as the vestibular nuclei, substantia nigra and cerebellum, which is normally associated with neuronal demise (Figures 4C,D). Furthermore, we also used RT-qPCR to examine the expression of the GFAP mRNA in the mouse cerebellum at P21 and P30. The differences in GFAP mRNA exression approached near significance (Supplementary Figure S2A).

**Figure 5:**
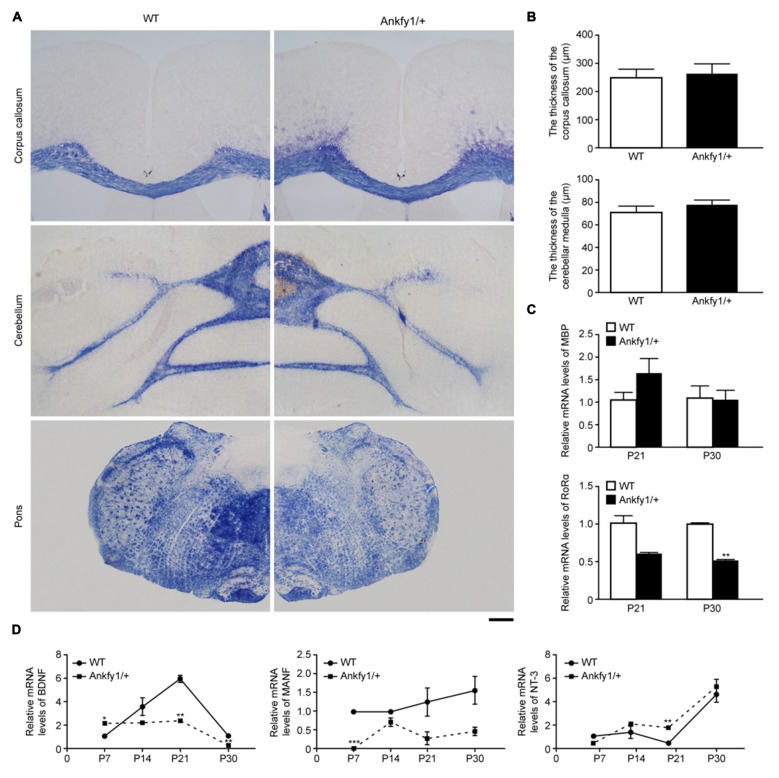
**Ankfy1/+ mice with normal myelin sheaths show reduced neurotrophic factor (NTF) mRNA expression. (A)** Eriochrome cyanine staining reveals similarly sized myelin sheaths in the corpus callosum, cerebellum and pons of Ankfy1/+ mice at 4 months of age. The scale bar represents 300 μm. **(B)** Quantitative assessment of the thickness of the corpus callosum and cerebellar medulla per image field (*n* = 12). **(C)** RT-qPCR was used to determine the levels of the myelin basic protein (MBP) and retinoic acid receptor-related orphan receptor α (RORα) mRNAs in the cerebellum of P21 and P30 WT and Ankfy1/+ mice. The relative abundance of the MBP mRNA was calculated by setting the value obtained for the control mice to 1, and Glyceraldehyde 3-phosphate dehydrogenase (GAPDH) served as an internal control. **(D)** Brain-derived neurotrophic factor (BDNF), mesencephalic astrocyte-derived neurotrophic factor (MANF) and neurotrophin-3 (NT-3) mRNA expression levels in the cerebellum of WT and Ankfy1/+ mice were analyzed using RT-qPCR. The mRNA expression levels were normalized to the GAPDH mRNA expression levels (*n* = 4, *p < 0.05, **p < 0.01, ***p < 0.001). The data represent means ± SEM.

Demyelination is observed in some types of cerebellar ataxias, such as SCA2 (Estrada et al., 1999) and Friedreich’s ataxia (Koeppen and Mazurkiewicz, 2013). We used Eriochrome cyanine staining to evaluate myelin levels in the cerebellum, pons and corpus callosum and determine whether demyelination occurred in Ankfy1/+ transgenic mice. However, no differences were observed (Figures 5A,B). In addition, we examined the expression of the MBP mRNA in the cerebellum at P21 and P30 for further validation. No differences were observed in the MBP mRNA levels between the two groups (Figure 5C, top). In addition, we also examined changes in the levels of the RORα mRNA, which is predominantly expressed in Purkinje cells in the cerebellum (Serra et al., 2006). At both P21 and P30, RORα mRNA expression levels were lower in Ankfy1/+ mice than in WT mice (Figure 5C, bottom). The change was more obvious at P30.

### Ankfy1 Disrupts Growth Factors in the Cerebellum

Three major classes of NTFs regulate neuronal development, survival and maintenance: conventional NTFs, such as BDNF, NT-3 and neurotrophin-4 (NT-4); the cerebral dopamine neurotrophic factor (CDNF)-MANF family; and the glial-derived neurotrophic factor (GDNF) family. Several studies have already identified protective roles for BDNF or MANF in preventing Purkinje cell degeneration (Schwartz et al., 1997; Yang et al., 2014) and for NT-3 in promoting Purkinje cell proliferation (Lindholm et al., 1993). However, researchers have not extensively determined whether the loss of Ankfy1 affects NTFs. We monitored NTF activity in the cerebella of 7-, 14-, 21- and 30-day-old mice by measuring BDNF, MANF and NT-3 mRNA levels. BDNF mRNA expression levels began increasing on P7 in WT mice and peaked on P21, but did not follow this trend in Ankfy1/+ mice, which exhibited significantly lower BDNF mRNA levels on P21 and P30 (Figure 5D, left). MANF mRNA expression was almost undetectable in Ankfy1/+ mice on P7. Furthermore, the MANF mRNA was expressed at higher levels in WT mice than in Ankfy1/+ mice at each time point, but the differences were not significant (Figure 5D, middle). We also used RT-qPCR to examine the expression of the NT-3 mRNA and observed a different trend compared with the BDNF and MANF mRNAs. NT-3 mRNA expression began to increase on P7 in Ankfy1/+ mice and surpassed the expression observed in WT mice beginning on P14. A significant difference between WT and Ankfy1/+ mice was observed at P21 (Figure 5D, right).

### Changes in the Morphology of Purkinje Cells from Ankfy1/+ Mice *In Vitro*

**Figure 6:**
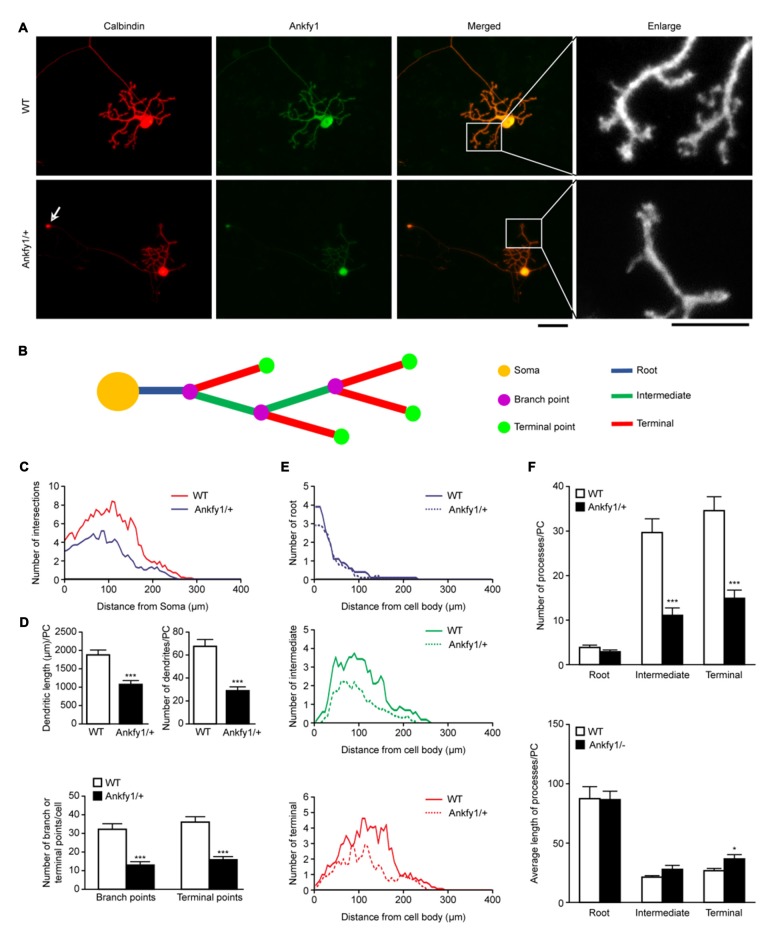
**Purkinje cell dendritic growth in WT and Ankfy1/+ cerebellar primary cell cultures. (A)** Representative images of Purkinje cells in primary cell cultures at DIV14 stained by calbindin and Ankfy1 antibody. Arrows indicate the circular expansion of the axon in the Ankfy1/+ neuron. Cultures were photographed by microscopy for dendrite tracing. The scale bars represent 50 μm (left) and 25 μm (right). **(B)** Schematic of Sholl analysis showed parameters as in panel **(A)**. **(C)** Total Sholl curves showed numbers of intersections of different distances from the Purkinje cell soma. Peak values positively correlated with dendritic density. **(D)** Quantitative analyses showed total dendritic length or amount and total branch or terminal point numbers per Purkinje cell (*n* = 10, ***p < 0.001). **(E)** Sholl analysis showed dendritic numbers within each process (root, intermediate and terminal) in WT and Ankfy1/+ cerebellar primary Purkinje cell cultures. Each curve area represented the average value of each process. **(F)** Average number and dendritic length of root, intermediate and terminal processes per cell (*n* = 10, *p < 0.05, ***p < 0.001). The data represent the means ± SEM.

A careful examination of the various cultured Purkinje cell populations from WT and Ankfy1/+ mice revealed specific defects in mutant Purkinje cells. As shown by calbindin or Ankfy1 immunostaining, Purkinje cells obtained from control mice exhibited a highly stereotypic dendritic bgranching pattern of 2–3 root-order branches emerging from the cell soma, followed by intermediate-order and terminal branches, with spiny branchlets observed at the intermediate level or above at DIV14 (Figure 6A and ideograph in Figure 6B). In contrast, Ankfy1/+ mutant Purkinje cells often possessed fewer intermediate or terminal dendritic branches at DIV14, and abundant distal spiny branchlets emerged from the terminal dendritic branches (Figure 6A). Calbindin-immunopositive axons of Purkinje cells from Ankfy1/+ mice showed circular expansions; more importantly, the Ankfy1/+ Purkinje cells displayed a significant increase in the number of axonal circular expansions compared with the Purkinje cells from the WT mice (Figure 6A, arrow and Supplementary Figure S2B). WT Purkinje cells also displayed more branches than Ankfy1/+ neurons at the same distance from the soma (Figure 6C), particularly in distal processes (Figure 6E). The total length and number of dendrites were clearly decreased in Ankfy1/+ Purkinje cells (Figure 6D, top). Moreover, the number of all branch points and terminal points differed significantly between Purkinje cells from WT and Ankfy1/+ mice (Figure 6D, bottom). We next analyzed the branch numbers and average branch lengths of different processes. As shown in Figure 6F (top), a large fraction of WT Purkinje cells had many more intermediate and terminal collaterals than the majority of collaterals observed in Ankfy1/+ Purkinje cells. However, the terminal branches from Ankfy1/+ Purkinje cells were longer (Figure 6F, bottom). Thus, Ankfy1 plays a role in Purkinje cell dendrite development. The morphological changes observed in Ankfy1/+ neurons may occur during the early stage of Purkinje cell death.

### Ankfy1 Deletion Promotes Apoptosis without Affecting Proliferation

**Figure 7:**
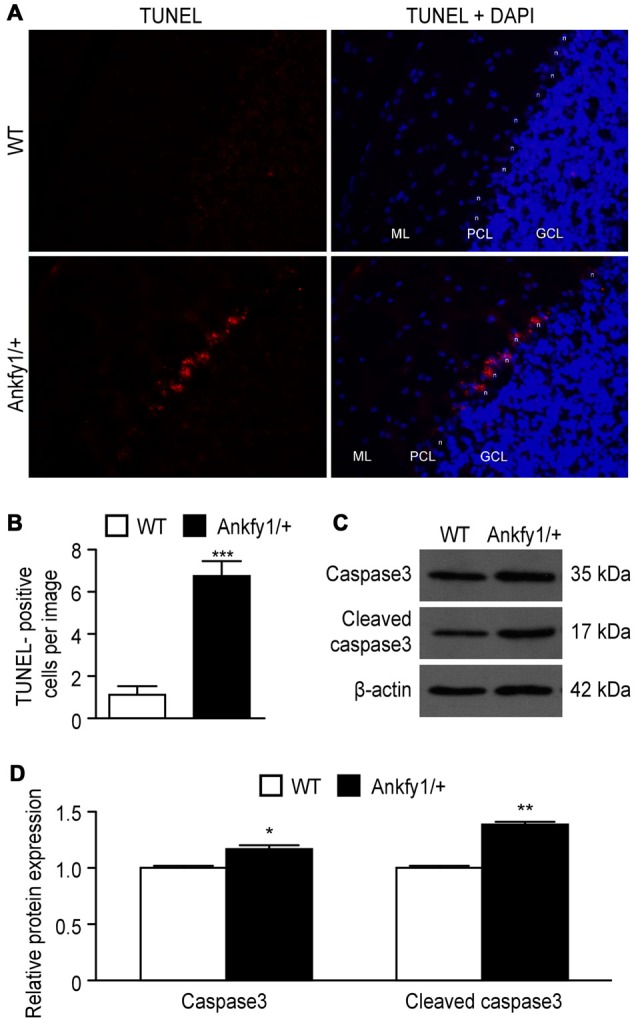
**Activation of apoptosis in Ankfy1/+ mice. (A)** Detection of apoptotic Purkinje cells using the TUNEL assay. We use “n” to represent the Purkinje cell nucleus, “ML” to represent the molecular layer, “PCL” to represent the Purkinje cell layer and “GCL” to represent the granule cell layer. The scale bars represent 50 μm. **(B)** Quantitative analyses showing the average number of TUNEL-positive cells per image (*n* = 5, ***p < 0.001). **(C)** Western blotting confirmed the expression of caspase3 and cleaved caspase3 in cerebellum of WT and Ankfy1/+ mice. **(D)** Quantitative analysis showed a significant increase in the levels of caspase3 and cleaved caspase3 in Ankfy1/+ mice (*p < 0.05, **p < 0.01).

According to several studies, Purkinje cell loss is thought to be caused by apoptosis in the cerebellum. However, researchers have not conclusively determined whether the Ankfy1 mutation affects apoptosis. In comparison to 6-month-old WT mice, staining with TUNEL, a specific stain for DNA breaks in both necrotic and apoptotic cells, revealed that the 6-month-old Ankfy1/+ mice exhibited severe DNA breaks in Purkinje cells (Figures 7A,B). We used western blots to examine the expression of the caspase3 and cleaved caspase3 proteins, an important effector caspase, to assess whether Ankfy1 influences the levels of apoptosis. Higher levels of caspase3 and cleaved caspase3 were detected in the cerebellum of Ankfy1/+ adult mice than in WT mice (Figures 7C,D).

**Figure 8:**
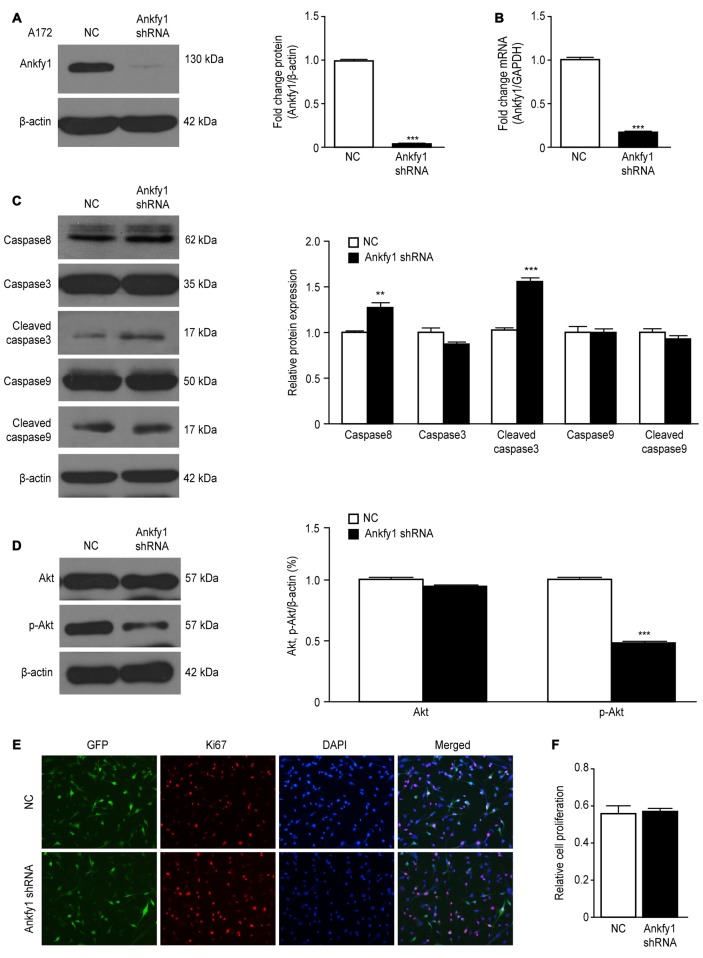
**Lentiviral knock-down of Ankfy1 expression in A172 cells promotes apoptosis. (A)** A172 cells were harvested 48 h after transfection with shRNAs, and levels of the Ankfy1 protein were analyzed using western blotting. β-actin was used as a loading control. Protein band intensities were normalized to β-actin expression levels (***p < 0.001). **(B)** Total RNA was extracted and subjected to RT-qPCR analysis to detect the expression of the Ankfy1 mRNA (***p < 0.001). **(C, D)** Western blots **(C)** and quantitation (**D**, **p < 0.01, ^***^p < 0.001) of the levels of the apoptotic proteins caspase3, cleaved caspase3, caspase8, caspase9 and cleaved caspase9. The levels of the β-actin protein are shown as an internal control. **(D)** Western blotting confirmed the expression of Akt and p-Akt in negative control (NC) shRNA- and Ankfy1 shRNA-transfected A172 cells. The quantitative analysis (right) showed that the Ankfy1 shRNA significantly decreased p-AKT expression (***p < 0.001). **(E,F)** Immunofluorescence staining showing similar numbers of Ki67-positive nuclei in NC shRNA- and Ankfy1 shRNA-transfected cells. The scale bar represents 100 μm. The graph of Figure 7E shows the percentage of cells with Ki67-positive nuclei. The data are presented as the means ± SEM of three independent experiments.

If a decreased Ankfy1 level in the mouse cerebellum contributed to Purkinje cell degeneration in an ARSACS mouse model, then decreased Ankfy1 expression should mimic this pathological change *in vitro*. We generated a lentiviral Ankfy1 shRNA vector in which the Ankfy1 shRNA was expressed under the control of the U6 promoter to test this hypothesis. A172 cells were transfected with either pLKO.1/Ankfy1 shRNA or the NC shRNA for 48 h. Cells expressing the Ankfy1 shRNA expressed significantly lower levels of the Ankfy1 mRNA and protein (Figures 8A,B) and shorter neurites (data not shown). As shown in Figure 8C, the levels of some apoptosis markers, such as caspase3, caspase8, caspase9, cleaved caspase3 and cleaved caspase9, were measured in lentiviral shRNA-transfected A172 cells. Suppression of Ankfy1 expression significantly increased the levels of caspase8 and cleaved caspase3 (Figure 8C). In addition, we detected the expression of the Akt and phosphorylated Akt proteins and showed that the down-regulation of Ankfy1 expression distinctly inhibited Akt phosphorylation in A172 cells (Figure 8D). Ki67 staining, a specific and sensitive method, was performed to determine the role of Ankfy1 in promoting cell growth. As shown in Figures 8E,F and Supplementary Figure S2C, Ki67-positive A172 and SH-SY5Y cells were observed in both the Ankfy1 and NC shRNA groups, although significant differences were not observed between these two different cell lines.

## Discussion

As shown in this study, Ankfy1 is required for the survival of cerebellar Purkinje cells. Ankfy1/+ mutant mice show severe ataxia and a substantial loss of Purkinje cells due to increased apoptosis during a period of programmed cell death (PCD). The Ankfy1 protein is ubiquitously expressed in various tissues and in the CNS (Kuriyama et al., 2000). Using *in situ* hybridization, the Ankfy1 mRNA was specifically expressed in cerebellar Purkinje cells and in the gray matter of the spinal cord. When we used gene-trap vectors to delete the Ankfy1 gene at exon 5, we did not generate Ankfy1 KO mice. Since the birth rates of WT and heterozygous mice fit the expected Mendelian ratios, Ankfy1 KO was confirmed to be lethal. The prominent motor behavioral abnormalities were consistent with the abnormalities described as clinical and pathological features of ARSACS (Schöls et al., 2004). However, we did not analyze the influence of gender on phenotype. Both male and female heterozygous mice had similar phenotypes, as indicated by motor behavior test values that followed the same trend over time. Similar to most patients with ARCAs, patients with ARSACS exhibit early-onset signs of spasticity in the lower limbs. These cerebellar disorder signs become apparent between the ages of 12 and 24 months, when the patients begin to walk (Bouslam et al., 2007; Shukla et al., 2011; Liu et al., 2015; van Schil et al., 2015; Nguyen et al., 2016). Similar to patients with ARSACS, the earliest observable motor deficit in Ankfy1/+ mice was observed at approximately 20 days of age, and the first measurable abnormal motor behavior was observed at 30 days using rotarod tests.

Moreover, Ankfy1/+ mutant mice displayed a severe loss of Purkinje cells starting at 60 days old. Although Purkinje cell death is a primary feature of motor disease, intriguingly, the ataxic gait phenotype was observed early in life without extensive Purkinje cell death. This finding raises important questions about the mechanisms by which Purkinje cells impact movement. Focusing our attention on this question, we used primary cultured Purkinje cells *in vitro* to mimic the phenotypic characteristics of Ankfy1/+ mutant mice *in vivo*. The dendritic trees of the mutant mice exhibited less arborization, and the size and number of dendritic branch points were reduced, with a substantial decrease in the number of terminal processes. Interestingly, circularly expanded axons were observed in the primary cultured Purkinje cells from the Ankfy1/+ mice; however, whether the expansion represents axonal swelling or the growth cone remains to be determined. Axonal swelling is a feature of the axonal dystrophy and degeneration that is associated with many CNS disorders, such as Alzheimer’s disease (Calkins et al., 2011), Parkinson’s disease (Orimo et al., 2008) and multiple sclerosis (Bjartmar et al., 2003). Axon swelling has been shown to precede, and sometimes cause, neuronal death in several disorders. Varicosities or spheroids are formed along the axons, preferentially at nodes of Ranvier, by focal blockade of anterograde axonal transport from the soma (Coleman, 2005). Based on these results, cell morphological changes caused by reduced Ankfy1 expression, occur much earlier than the onset of neuronal death, clearly indicating that these changes do not occur secondary to neurodegeneration. Changes in Purkinje cell morphology may underlie the motor coordination deficits. Taken together, these results support the hypothesis that the motor phenotypes of Ankfy1/+ mutants are primarily due to pathological changes in Purkinje cells.

Oligodendrocytes provide myelin for both rapid impulse conduction and long-term support for axons in the nervous system (Griffiths et al., 1998). Using RT-qPCR to examine the mRNA levels of MBP, the second most abundant CNS myelin protein, in the cerebella of 21- and 30-day-old Ankfy1/+ mice, 21-day-old but not 30-day-old mice displayed increased MBP mRNA levels, although a substantial increase was not observed. However, no major changes in the myelin volume of the cerebellar medulla, corpus callosum or pons were observed by Eriochrome cyanine staining. Thus, the axonal swelling observed in primary cultured Purkinje cells of Ankfy1/+ appeared to be related to the absence of Ankfy1 and not to abnormal myelination. As most mutant proteins linked to neurodegenerative diseases exhibit a broad expression pattern, their expression at locations other than vulnerable neurons, particularly within glial cells, which form intimate contacts with neurons, contributes to disease mechanisms. Mutant proteins within glial cells drive toxicity in neighboring neurons either by the release of toxic components or by a mutant-mediated reduction in one or more neuronal support functions (Lobsiger and Cleveland, 2007). Astrocytes were also assessed in the present study. GFAP was expressed in the white matter glia and Bergmann glia in the cerebellum, and its expression by reactive astrocytes is perhaps the most well-known hallmark of reactive astrocytes and gliosis. Because of the use of horizontal slices, the typical Bergmann cell morphology was partially destroyed. However, an increase in GFAP immunostaining was observed in specific areas, such as the cerebellum, vestibular nuclei and substantia nigra, in the Ankfy1/+ mice. Consistent with this result, the RT-qPCR analysis showed a higher expression level of the GFAP mRNA in the cerebella of 21- and 30-day-old Ankfy1/+ mice, and the increases approached near significance. These findings are consistent with the pathological findings reported in patients with neurodegenerative diseases (Di Giorgio et al., 2007; Nagai et al., 2007) that involve gliosis.

Mutant Ankfy1 affects BDNF, NT-3 and MANF expression in Ankfy1/+ mice. Classically, NTFs are small secreted proteins that regulate neuronal survival, differentiation and maturation, as well as neurite growth and branching, by binding and activating their cognate receptors. During periods of PCD in development, NTFs prevent apoptosis and regulate the number of neurons innervating the target tissue (Lindholm and Saarma, 2010). Only neurons that receive sufficient trophic support and contact the target tissue with synapses survive, whereas other neurons undergo apoptosis. We provide several lines of evidence supporting the roles of MANF and BDNF in Purkinje cell degeneration in ARSACS. First, the earliest discovered and the best studied NTFs include BDNF and NT-3, which are abundantly expressed in granule cells in the cerebellum to regulate neuronal survival and plasticity (Sadakata et al., 2007). Second, relatively high MANF levels were detected in cerebellar Purkinje cells (Yang et al., 2014), which are believed to exert their neuroprotective effects both extracellularly by binding to a currently unidentified receptor (Voutilainen et al., 2009), and intracellularly by inhibiting apoptosis (Hellman et al., 2011). Finally, the expression of the BDNF and MANF mRNAs, but not NT-3 mRNA, was reduced in cerebellum samples from Ankfy1/+ mice. Thus, low levels and dysfunction of Anfky1 can decrease BDNF and MANF levels, contributing to the progression of Purkinje cell degeneration in ARSACS via a similar mechanism to the loss of function reported for other neurodegenerative diseases (Lim et al., 2008; Blurton-Jones et al., 2009).

Another important finding of our study is that Ankfy1/+ mutant Purkinje cells contain DNA strand breaks, and Ankfy/+ mice exhibit increased expression of the caspase3 and cleaved caspase3 proteins, a key effector caspase, in the adult cerebellum. We transfected A172 cells with an Ankfy1 shRNA to further investigate the molecular mechanism of Purkinje cell death in ARSACS and found that Ankfy1 increased the levels of caspase8 and cleaved caspase3 but did not affect proliferation, suggesting that apoptosis may be the mechanism by which reduced Ankfy1 expression induced cell death. Another important finding of our study is that decreasing Ankfy1 expression in A172 cells suppressed Akt phosphorylation. The effects of phosphatidylinositol 3-kinase (PI3-K) on cell survival might be mediated by the protein kinase Akt, which is activated by a number of NTFs, including BDNF and NT-3, through a PI3-K-dependent mechanism (Ness et al., 2002; Islam et al., 2009). PI3-K activation is required for BDNF-induced hippocampal neuronal proliferation (Luo et al., 2015). These observations, together with our present findings, suggest that Akt may function downstream of Ankfy1-mediated BDNF and MANF secretion and dendritic differentiation in Purkinje cells. However, further studies are required to determine whether Ankfy1 has important roles in the internalization or trafficking of these NTFs receptors.

In summary, the most obvious deficit in Ankfy1/+ mice was a reduction in motor coordination, which indicates cerebellar dysfunction. In the current study, we focused on these phenotypes and showed that Ankfy1 is required for normal cerebellar Purkinje cell development. Ankfy1 is also expressed in several other regions of the brain and may mediate diverse functions.

## Author Contributions

MD and ZL designed the study and wrote the article. All authors contributed to the analysis and interpretation of the data presented in this study. In addition, all authors edited and approved the manuscript.

## Funding

This work was supported by grants from Health and Family Planning Commission of Hubei Province scientific research project (WJ2015MA007) and Wuhan Science and Technology Bureau scientific research project (2015060101010047).

## Conflict of Interest Statement

The authors declare that the research was conducted in the absence of any commercial or financial relationships that could be construed as a potential conflict of interest.

## Supplementary Material

The Supplementary for this article can be found online at: http://journal.frontiersin.org/article/10.3389/fnmol.2017.00121/full#supplementary-material

Supplementary Figure 1 **An overview of the phenotype and morphology and hemeatoxylin and eosin (H&E) staining of the cerebellum of Ankfy1/+ mice. (A)** Representative appearance of the brains from Ankfy1/+ mice and wild-type (WT) mice at 52 weeks of age. **(B)** Photographs of Ankfy1/+ mice at 12 weeks of age showing the clasping and kyphosis phenotypes. **(C)** H&E staining (20×) of the cerebella from Ankfy1/+ and WT mice revealed Purkinje cell loss in the transgenic mice at the age of P60. Purkinje cell bodies are outlined in white to delineate the Purkinje cells. Scale bar: 50 μm. **(D)** Quantification and analysis of the number of Purkinje cells and thickness of the molecular layer in the images shown in **(C)** indicated that the cerebellum in Ankfy1/+ mice exhibit a significant loss of Purkinje cells beginning at P60, but no changes in the thickness of the molecular layer were observed compared with the control cerebellum (****p* < 0.001). The data are presented as the means ± SEM.

Supplementary Figure 2 **Glial fibrillary acidic protein (GFAP) mRNA expression, quantification of circular expansions of axon in primary cultured Purkinje cells, and Ki67 staining of SH-SY5Y cells from the negative control (NC) and Ankfy1 shRNA groups. (A)** RT-qPCR was used to detect the levels of the GFAP mRNA in the cerebella of P21 and P30 WT and Ankfy1/+ mice. The relative abundance of the GFAP mRNA was calculated by setting the value of the control mice to 1, and glyceraldehyde 3-phosphate dehydrogenase (GAPDH) served as an internal control (*p* = 0.0614 in P21, *p* = 0.0940 in P30). **(B)** Average number of circular expansions of axon per Purkinje cell was calculated (*n* = 10., ****p* < 0.001). **(C)** Significant differences were not observed between the NC shRNA-transfected and Ankfy1 shRNA-transfected cells. The scale bar represents 100 μm. **(D)** Quantification of the percentage of Ki67-positive nuclei in the Ankfy1 shRNA-transfected SH-SY5Y cells. The data are presented as the means ± SEM of three independentg experiments.

## Abbreviations

ADCAs: autosomal dominant cerebellar ataxias
ARCAs: autosomal recessive cerebellar ataxias
ARSACS: autosomal recessive spastic ataxia of Charlevoix-Saguenay
BDNF: brain-derived neurotrophic factor
CNS: central nervous system
H&E: hemeatoxylin and eosin
MANF: mesencephalic astrocyte-derived neurotrophic factor
MBP: myelin basic protein
NC: negative control
NT-3: neurotrophin-3
NT-4: neurotrophin-4
NTFs: neurotrophic factors
PCD: programmed cell death
RORα: retinoic acid receptor-related orphan receptor α
WT: wild-type.
