# Supplementary figures and images for: Purkinje Cell Degeneration and Motor Coordination Deficits in a New Mouse Model of Autosomal Recessive Spastic Ataxia of Charlevoix-Saguenay
Source: Front Mol Neurosci. 2017 May 1;10:121. doi: 10.3389/fnmol.2017.00121 (PMC5440006; doi:10.3389/fnmol.2017.00121)

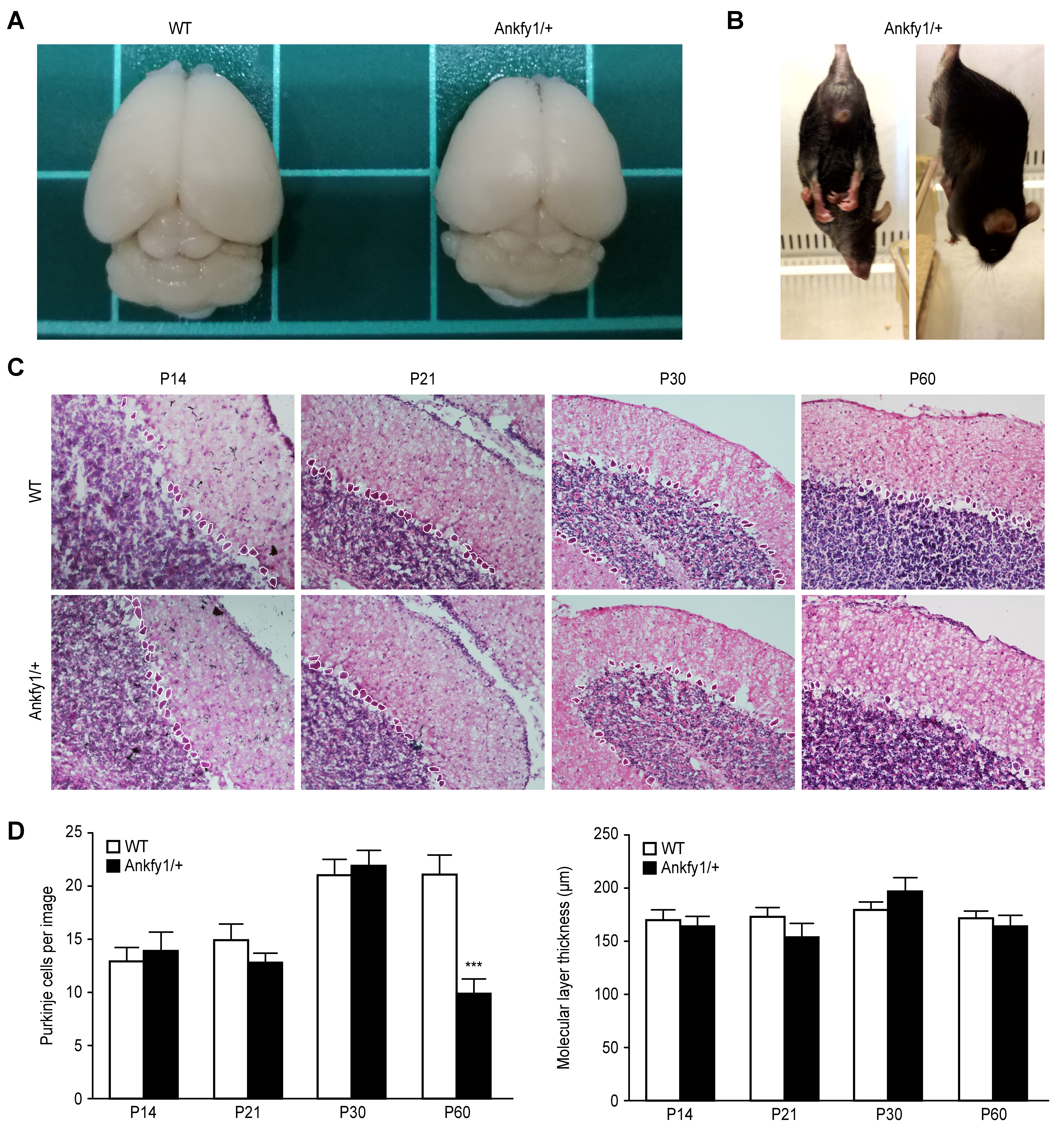

Supplement: Supplementary Figure 1 — An overview of the phenotype and morphology and hemeatoxylin and eosin (H&E) staining of the cerebellum of Ankfy1/+ mice. (A) Representative appearance of the brains from Ankfy1/+ mice and wild-type (WT) mice at 52 weeks of age. (B) Photographs of Ankfy1/+ mice at 12 weeks of age showing the clasping and kyphosis phenotypes. (C) H&E staining (20×) of the cerebella from Ankfy1/+ and WT mice revealed Purkinje cell loss in the transgenic mice at the age of P60. Purkinje cell bodies are outlined in white to delineate the Purkinje cells. Scale bar: 50 μm. (D) Quantification and analysis of the number of Purkinje cells and thickness of the molecular layer in the images shown in (C) indicated that the cerebellum in Ankfy1/+ mice exhibit a significant loss of Purkinje cells beginning at P60, but no changes in the thickness of the molecular layer were observed compared with the control cerebellum (***p < 0.001). The data are presented as the means ± SEM. [file Image_1.tif]

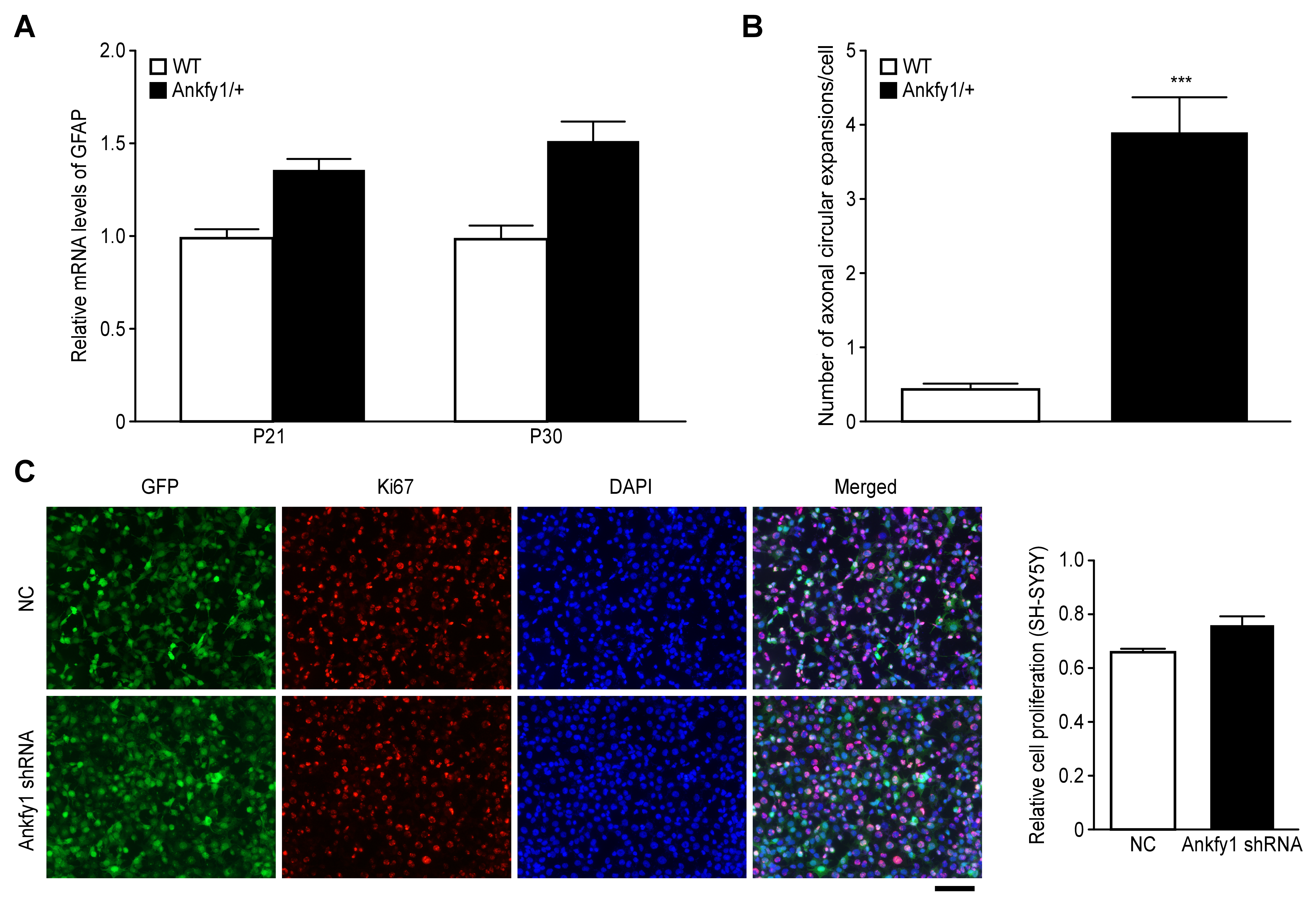

Supplement: Supplementary Figure 2 — Glial fibrillary acidic protein (GFAP) mRNA expression, quantification of circular expansions of axon in primary cultured Purkinje cells, and Ki67 staining of SH-SY5Y cells from the negative control (NC) and Ankfy1 shRNA groups. (A) RT-qPCR was used to detect the levels of the GFAP mRNA in the cerebella of P21 and P30 WT and Ankfy1/+ mice. The relative abundance of the GFAP mRNA was calculated by setting the value of the control mice to 1, and glyceraldehyde 3-phosphate dehydrogenase (GAPDH) served as an internal control (p = 0.0614 in P21, p = 0.0940 in P30). (B) Average number of circular expansions of axon per Purkinje cell was calculated (n = 10., ***p < 0.001). (C) Significant differences were not observed between the NC shRNA-transfected and Ankfy1 shRNA-transfected cells. The scale bar represents 100 μm. (D) Quantification of the percentage of Ki67-positive nuclei in the Ankfy1 shRNA-transfected SH-SY5Y cells. The data are presented as the means ± SEM of three independentg experiments. [file Image_2.tif]
